# Viral Manipulation of Host Inhibitory Receptor Signaling for Immune Evasion

**DOI:** 10.1371/journal.ppat.1005776

**Published:** 2016-09-01

**Authors:** Eugenia Z. Ong, Kuan Rong Chan, Eng Eong Ooi

**Affiliations:** 1 Experimental Therapeutics Centre, Agency for Science, Technology, and Research, Singapore; 2 Programme in Emerging Infectious Diseases, Duke-NUS Medical School, Singapore; 3 Department of Microbiology and Immunology, Yong Loo Lin School of Medicine, National University of Singapore, Singapore; 4 Saw Swee Hock School of Public Health, National University of Singapore, Singapore; 5 Singapore-MIT Alliance in Research and Technology, Infectious Diseases Interdisciplinary Group, Singapore; Columbia University, UNITED STATES

## Introduction

The immune system has evolved pairs of activating and inhibitory receptors that modulate the magnitude of immune responses, enabling the maintenance of immune homeostasis. Inhibitory signaling dampens the immune response, which prevents inflammatory damage to the host. It has now become increasingly clear that viruses have evolved means of exploiting the inhibitory signaling pathways of the immune system in order to blunt the responses that would otherwise abrogate infection. Recent evidence demonstrates how viruses exploit inhibitory receptors both for host cell entry and to down-regulate antiviral responses for enhanced viral pathogenesis. Both acute and chronic viral infections also induce expression of intermediates of inhibitory signaling for improved odds of survival within the intracellular environment. This review highlights and synthesizes from recent findings how medically important viruses exploit the inhibitory pathways that maintain immune homeostasis for successful human infection.

## The Yin and Yang of the Immune System

The rapid initiation and timely termination of the immune response are coordinated by paired receptors expressed on immune cells. Paired receptors consisting of activating and inhibitory receptors recognize self and non-self ligands. They are essential for maintaining self-tolerance, mounting an immune response during infection, and modulating the intensity of the response to prevent autoimmunity and inflammatory damage to bystander cells [1]. Activating receptors recruit adaptor molecules containing immunoreceptor tyrosine-based activation motifs (ITAMs) or bear ITAMs in their cytoplasmic tails for signaling. Upon ligation and clustering of activating receptors, ITAMs are phosphorylated by Src family kinases, which create docking sites for Src homology 2 (SH2)-domain containing kinases like spleen tyrosine kinase (Syk) and zeta chain-associated protein kinase 70 (ZAP-70) [1]. These kinases phosphorylate downstream substrates and form receptor-proximal signaling complexes that drive phagocytosis, cellular activation, and pro-inflammatory responses. To achieve immune homeostasis, inhibitory receptors containing one or several immunoreceptor tyrosine-based inhibitory motifs (ITIMs) in the cytoplasmic tail are employed [2]. Ligation of inhibitory receptors leads to Src family kinase-mediated ITIM phosphorylation and recruitment of SH2-domain containing cytoplasmic phosphatases like SH2-containing phosphatase 1 (SHP-1), SHP-2, and SH2-containing inositol phosphatase 1 (SHIP-1) [2]. Dephosphorylation of downstream signaling effector molecules results in down-regulation of immune responses and maintenance of peripheral self-tolerance.

Although activating and inhibitory signals are integrated for immune homeostasis, they might not contribute proportionately to signaling output due to dominant inhibitory signaling, as exemplified by natural killer (NK) cell responses [3]. NK cells are innate lymphocytes that have a role in early control of infection and tumorigenesis, and recent studies have provided insight on how paired receptor signaling facilitates NK cell effector functions. Binding of viral proteins or cellular stress-induced proteins to activating receptors on NK cells triggers the release of cytolytic granules to eliminate infected or transformed cells [3]. Conversely, inhibitory receptors on NK cells bind major histocompatibility complex class I (MHC-I) molecules, which induces self-tolerance and protects bystander cells against inflammatory damage. Paradoxically, inhibitory signaling also maintains NK cell responsiveness, as NK cells devoid of inhibitory receptors are hyporesponsive [4]. Interaction of inhibitory receptors with MHC-I molecules increases NK cell responsiveness through a dynamic process known as “licensing,” which in turn sensitizes NK cells to signaling from activating receptors. Evidently, signaling from paired receptors is not always integrated in a simple balance and thus serves as a caveat when interpreting the functional outcome of receptor engagement by pathogen-derived ligands or signaling intermediates.

The complexity of signal integration is further underscored by how signaling outcome is tuned by viral manipulation of inhibitory signaling. Coevolution of viruses and their hosts has resulted in viruses acquiring strategies for attenuating immune responses to favor viral replication and disease in humans. Viruses are known to utilize inhibitory receptors for host cell entry, which also initiate inhibitory signaling to down-regulate antiviral responses for enhanced viral replication. The recent use of quantitative temporal viromics, which profiles proteomic changes in viral and host cell proteins over time, has demonstrated how viruses also dramatically alter the expression of cell surface proteins to counter the host cell’s defenses [5]. Finally, viruses that cause chronic diseases in humans utilize inhibitory signaling to impose T cell exhaustion, which greatly hampers response to disease treatment. We highlight recent evidence of how viruses exploit inhibitory signaling, leading to enhanced viral pathogenesis and human disease.

## Viral Interaction with Inhibitory Receptors during Host Cell Entry Down-Regulates Antiviral Responses

The viral life cycle starts with host cell entry, which involves direct fusion with cell membrane or ligating an appropriate receptor to trigger endocytosis, pinocytosis, or macropinocytosis. Use of inhibitory receptors during host cell entry could thus simultaneously initiate inhibitory signaling to dampen the immune response for enhanced viral replication (Table 1).

**Table 1:** Viruses engage inhibitory receptors to down-regulate antiviral responses.

Virus	Inhibitory receptor	Functional importance	Reference
DENV	CD300a	Attachment factor to enhance infection	[8]
	LILRB1	Blocks up-regulation of ISGs to enhance viral replication	[10]
HIV-1	DCIR	Attachment factor to facilitate viral replication in DCs and CD4^+^ T cells	[14,15]
Measles (H)	SLAMF1	Inhibits TLR4-induced production of IL-12 in DCs	[19]
Measles vaccine strain, HHV-6, rAdV type 35	CD46	Protects infected cells from complement attack, inhibits production of IL-12 and IL-2, which suppresses activation of NK cells and T cells	[21–23]

CD300a is an inhibitory receptor that belongs to the CD300 family of transmembrane receptors. It binds cell surface phosphatidylserine and phosphatidylethanolamine (PE), which are exposed following increased levels of intracellular calcium during human immunodeficiency virus type 1 (HIV-1) and hepatitis C virus (HCV) infection [6]. Interaction between CD300a and its ligands results in SHP-1 recruitment for suppression of NK cell-mediated cytolysis in tumor cells [7] and inhibition of FcεRI-mediated mast cell activation [6]. CD300a also serves as an attachment factor for dengue virus (DENV) and enhances DENV infection in primary macrophages [8]. It is likely that CD300a recognizes PE on the virion surface, possibly acquired when DENV buds from the endoplasmic reticulum during replication. Because CD300a has been shown to inhibit toll-like receptor (TLR)-dependent inflammatory pathways [9], ligation of CD300a by DENV could inhibit or delay innate immune response against infection.

**Fig 1:**
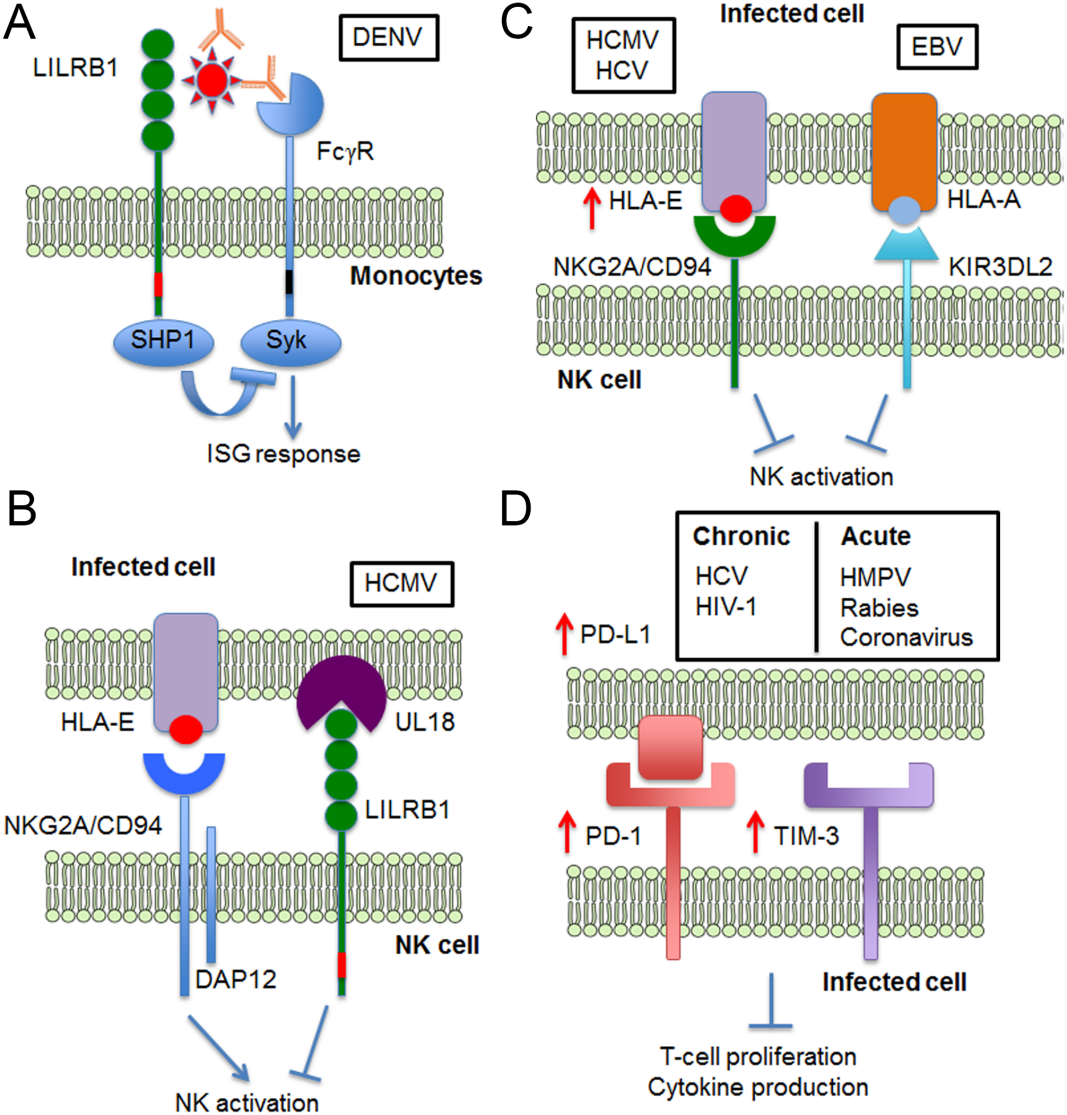
Viruses manipulate host inhibitory receptor signaling for immune evasion. A. Dengue virus (DENV) opsonized with sub- or non-neutralizing levels of antibodies is taken up via FcγRs on myeloid cells. By co-ligating inhibitory receptor LILRB1, antibody-opsonized DENV blocks FcγR-mediated up-regulation of interferon-stimulated genes (ISGs) to enhance viral replication. B. Human cytomegalovirus (HCMV) expresses UL18, an MHC class I homologue, which triggers LILRB1-mediated inhibitory signaling to limit antiviral effector functions and NK cell lysis. Peptide derived from UL40 also increases HLA-E expression, and the increased interaction between HLA-E and CD94/NKG2A inhibits NK cell lysis. C. HCMV, hepatitis C virus (HCV), and Epstein Barr virus (EBV) produce peptides that stabilize the expression of HLA-E and HLA-A. This increases interaction with inhibitory receptors like CD94/NKG2A and KIR3DL2 to inhibit NK cell activation. D. Expression of inhibitory receptors PD-1 and TIM-3, as well as the PD-1 ligand. PD-L1 is up-regulated during chronic and acute viral infections. PD-1 and TIM-3 inhibitory signaling results in impaired T cell activation and T cell exhaustion, leading to viral persistence in chronic infections.

DENV is also known to ligate leukocyte immunoglobulin-like receptor B1 (LILRB1) [10], an inhibitory receptor expressed on monocytes, dendritic cells (DCs), and subsets of NK, B, and T cells [11]. Binding of LILRB1 to its natural ligand, major histocompatibility class I (MHC-I) molecules, leads to recruitment of SHP-1 and potentiates negative feedback mechanisms such as inhibition of B cell receptor signaling and inhibition of cell killing by NK and T cells [11]. During antibody-enhanced DENV infection, non- or sub-neutralizing levels of antibodies form immune complexes with DENV, which are taken up via activating FcγRs on myeloid cells [12]. This phenomenon, also known as antibody-dependent enhancement (ADE), is postulated to explain the heightened risk of severe dengue following secondary infection. Ligation of activating FcγRs by antibody-opsonized DENV leads to Syk and STAT-1 phosphorylation, activating transcription of interferon-stimulated genes (ISGs), known to inhibit viral replication [13]. Unlike its interaction with CD300a, co-ligation of LILRB1 does not alter the rate of viral entry. Instead, co-ligation of LILRB1 by DENV resulted in SHP-1 recruitment, dephosphorylation of Syk, and down-regulation of ISGs, which permits successful viral replication during ADE (Fig 1A) [10].

Besides modulating the intracellular environment of the infected cell, viruses also tap inhibitory signaling to alter cytotoxic cellular responses. Dendritic cell immunoreceptor (DCIR), an ITIM-containing C-type lectin, serves as an attachment factor for HIV-1. HIV-1 drives DCIR expression on CD4^+^ T cells, and binding of HIV-1 to DCIR promotes infection of DCs and CD4^+^ T cells [14,15]. Because endocytosed DCIR suppresses the production of IL-12 and IFN-α, this also inhibits antiviral activity against HIV-1 [16]. Furthermore, ITIM-dependent DCIR signaling was recently shown to modulate the release of exosomes containing DAP-3, a pro-apoptotic protein that induced apoptosis in bystander CD4^+^ T cells [17]. These findings collectively indicate that HIV-1 capitalizes on DCIR to enhance infection and trigger cytotoxic response against CD4^+^ T cells.

Viruses also exploit inhibitory signaling to curtail pattern recognition receptor signaling. Interaction of measles virus (MeV) hemagglutinin (H) with its cellular receptor, signaling lymphocytic activation molecule family 1 (SLAMF1), permits viral entry [18]. Interaction of SLAMF1 and H protein was found to inhibit TLR4-induced production of IL-12 in DCs [19]. This could account for the clinical observations of immunosuppression and enhanced susceptibility to opportunistic infections during MeV infection [20].

CD46 or membrane cofactor protein is a complement inhibitor that inactivates C3b and C4b. Interestingly, both the MeV vaccine strain and human herpesvirus 6 (HHV-6) binds CD46, which protects infected cells from complement attack [21,22]. It also inhibits IL-12 production by macrophages, suppressing NK cell and T cell activity [21,22]. Likewise, ligation of CD46 by recombinant adenovirus (rAdV) type-35 vectors also blocked IL-2 production and CD4^+^ T cell activation [23]. An important consideration in the use of rAdV for vaccine or gene delivery would thus involve ablation of binding to CD46 for inducing transgene-specific immunity. Evidently, the immunomodulatory role of CD46 and its ability to bridge the innate and adaptive immune response explain why it is such an attractive target for multiple viruses.

## Viral Evasion of NK Cell-Mediated Immunity

NK cells serve as sentinels of the immune response, expressing a repertoire of paired receptors that enables discrimination of healthy cells from infected or transformed cells. During viral infections, viruses have to strike a delicate balance of limiting MHC-I presentation of viral peptides and maintaining sufficient levels of MHC-I to avoid NK cell-mediated lysis [24]. By expressing viral proteins that serve as MHC-I mimics and modulation of MHC-I molecules, viral manipulation of inhibitory signaling on NK cells constitutes a key thrust of how viruses overcome host immunity (Table 2).

**Table 2:** Viral evasion of NK cell-mediated immunity.

Virus	Inhibitory signaling intermediate	Functional importance	Reference
HCMV	UL18 binds LILRB1	Limits antiviral effector functions, protects infected cells from NK cell lysis	[26]
	Peptide derived from UL40 increases HLA-E expression	Increased interaction between HLA-E and CD94/NKG2A inhibits NK cell lysis	[28,30]
HCV	HCV core protein stabilizes HLA-E	Increased interaction between HLA-E and CD94/NKG2A inhibits NK cell lysis	[29]
EBV	Peptide derived from EBNA-3A binds HLA-A	Increased interaction between HLA-A and KIR3DL2 inhibits NK cell lysis	[27]
HIV-1	Variant epitope from Vpu and Env binds KIR2DL2	Presentation on HLA-C allows infected cells to evade NK cell lysis	[33]
	Variant epitope from p24 Gag binds KIR2DL3	Viruses with high mutation rates could manipulate epitopes presented to bind inhibitory receptors	[34]
DENV	Peptide derived from NS1 binds KIR3DL1	Patients with marked activation of B57-NS1^+^ NK cells developed dengue hemorrhagic fever	[35]

HCMV expresses UL18, a MHC-I homologue which binds LILRB1 with >1,000-fold greater affinity relative to other MHC-I molecules [25]. Binding of UL18 to LILRB1 inhibits antiviral effector functions and protects HCMV-infected cells from NK cell-mediated cytolysis (Fig 1B) [26]. Likewise, Epstein Barr virus (EBV) expresses the viral protein EBNA-3A, which supplies peptides that bind certain HLA-A allotypes. These HLA-peptide complexes are recognized by the inhibitory receptor KIR3DL2, which prevents NK cell lysis of EBV-infected cells (Fig 1C) [27].

HCMV and HCV also suppress NK cell activation through the inhibitory CD94/NKG2A receptor complex. The HCV core protein and a nonameric peptide derived from HCMV UL40 glycoprotein stabilizes HLA-E expression on infected cells [28,29]. This mechanism facilitates the interaction between HLA-E and CD94/NKG2A, conferring resistance to NK cell lysis (Fig 1C) [28,30]. In contrast, HIV-1 Gag (capsid protein) presented on HLA-E was unable to interact with CD94/NKG2A receptor on NK cells, resulting in specific lysis of HIV-1 infected CD4^+^ T cells [31]. However, switching the asparagine residue to phenylalanine in the HIV-1 capsid peptide inhibited NK cell degranulation [31]. The requirement for peptide selectivity to fully engage inhibitory receptors on NK cells thus serves as an additional layer of immune regulation to challenge viral evasion [32].

Besides modulating MHC-I levels, viruses like HIV-1 can manipulate the types of epitopes presented on infected cells to bind inhibitory receptors on NK cells. Significant enrichment of HIV-1 polymorphisms in the region encoding the Vpu and Env proteins was detected in individuals with KIR2DL2^+^ NK cells [33]. Presentation of these variant peptides on HLA-C enhanced binding to KIR2DL2, inhibiting NK cell-mediated cytolysis. Separately, HIV-1 polymorphisms in p24 Gag presented in HLA-C*03:04 individuals enhanced binding with KIR2DL3 on NK cells [34]. Although the functional outcome of epitope selection by HIV-1 has not been determined on viral fitness or control of HIV-1 replication in patients, it signifies a novel strategy that viruses could adopt to escape NK cell-mediated immune surveillance.

Along with viruses that cause persistent infections in human hosts, viruses causing acute disease can also exploit the interaction between MHC-I molecules and inhibitory receptors for improved replication and dissemination. An HLA-B57-restricted epitope derived from DENV non-structural protein-1 (NS1) was found to interact with KIR3DL1 on NK cells [35]. Interestingly, there was marked activation of B57-NS1^+^ NK cells during a critical phase of illness in patients who developed dengue hemorrhagic fever [35]. Although this interaction suggests a strategy by DENV to evade NK cell-mediated immunity, functional studies remain to be conducted for a definitive conclusion.

## Manipulation of Inhibitory Signaling for Viral Persistence

Viruses that cause chronic diseases and persistent infection in their human hosts exploit inhibitory signaling to prevent viral clearance (Table 3). NK cells are widely recognized as innate immune effector cells and also produce cytokines like IFN-γ and TNF-α to activate DCs and T cells. Thus, inhibitory signaling on NK cells could down-regulate the adaptive immune response, suppressing viral clearance to drive chronic infection. Indeed, CD94/NKG2A was up-regulated in NK cells from chronic HCV-infected patients [36]. NK cells from these donors were deficient in activating DCs and produced IL-10 and TGF-β when cultured with hepatic cells expressing HLA-E [36].

**Table 3:** Viral manipulation of inhibitory signaling drives chronic and persistent infections.

Virus	Inhibitory signaling intermediate	Functional importance	Reference
HCMV	UL18 binds LILRB1	Protects infected cells from cytotoxic T cell lysis	[37]
HCV	HCV core protein drives PD-L1 expression	PD-L1 binds PD-1 resulting in T cell exhaustion and viral persistence	[40]
HIV-1	Up-regulation of PD-L1 and PD-1 during infection	IL-10 induction; impairs CD4^+^ T cell activation	[41–42]
HMPV, RSV, rabies, coronavirus	Up-regulation of PD-1 during infection	PD-1 inhibitory signaling results in T cell exhaustion during acute viral infections	[46–49]

Broad multitypic CD4^+^ and CD8^+^ T cell responses are critical for viral clearance and are undermined by immune exhaustion during chronic infection. During persistent HCMV infections, LILRB1 expression is elevated on HCMV-specific CD8^+^ cytotoxic T cells (CTLs) with differentiated effector memory phenotype [37]. In contrast, lower expression of LILRB1 was observed in CTLs specific for EBV and influenza virus [37]. LILRB1 binds the HCMV glycoprotein UL18 to inhibit antiviral activity in NK cells and monocytes. In this context, UL18-LILRB1 interaction may down-regulate HCMV-specific effector memory T cell responses, driving HCMV reactivation and prolonging HCMV infections. This thus highlights how viruses can manipulate LILRB1 to inhibit both innate and adaptive host responses.

In persistent HCV and HIV-1 infections, inhibitory receptors like programmed cell death protein 1 (PD-1) and T cell Ig and mucin domain-containing molecule 3 (TIM-3) are up-regulated on functionally impaired cytotoxic CD8^+^ T cells [38,39]. The HCV core protein up-regulates PD-L1 expression on Kupffer cells, which binds PD-1 to promote T cell dysfunction and development of viral persistence (Fig 1D) [40]. PD-1 and PD-L1 are up-regulated in monocytes and macrophages upon HIV-1 infection, inducing high levels of IL-10 that impair CD4^+^ T cell activation (Fig 1D) [41,42]. PD-L1^high^ infected cells also escaped from cytotoxic T cell killing and accumulated over the course of HIV-1 infection [43]. T cell exhaustion can arise from negative feedback on cytotoxic T cells via PD-1 inhibitory signaling. These observations demonstrate how viruses evade cytotoxic cellular responses and could explain how viral reservoirs are established during chronic viral infections. Reduced levels of PD-1 on CD4^+^ and CD8^+^ T cells correlate to the control of both HCV and HIV-1 replication, while blocking PD-1 enhanced T cell proliferation [44,45].

T cell exhaustion is not unique to chronic infections but could also rapidly develop in acute viral respiratory infections with human metapneumovirus (HMPV) [46] or respiratory syncytial virus (RSV) [47]. Likewise, acute neurological infections with rabies [48] or coronavirus [49] also produce similar effects. These infections are driven by increased PD-1 expression on CD8^+^ T cells, rapidly inducing a transcriptional state synonymous with T cell exhaustion (Fig 1D). Antigen-dependent up-regulation of other inhibitory receptors such as TIM-3, lymphocyte activation gene 3 (LAG-3), and 2B4 was also observed with HMPV infection [46]. These inhibitory receptors thus contribute to T cell exhaustion during acute viral infections by working in concert with PD-1 signaling.

## Concluding Remarks

A substantial body of work has now refined our mechanistic understanding of how various medically important viruses manipulate inhibitory signaling for survival within the host cell. Given that members of a virus family share many conserved structural and non-structural proteins, it is plausible that viral strategies to manipulate inhibitory signaling could be relevant to a broader range of viruses than those discussed here. Understanding how viruses exploit inhibitory signaling could lead to rationally designed interventions that interrupt these critical virus–host interactions. The potential of an anti-PD-L1 antibody in reducing viral reservoirs in HIV-1 patients was recently evaluated in a clinical trial (NCT02028403). We anticipate that a combination of therapies targeting critical steps of the viral life cycle and boosting different arms of the immune response could provide recourse for both acute and persistent viral infections.
